# Ubiquitin recognition by FAAP20 expands the complex interface beyond the canonical UBZ domain

**DOI:** 10.1093/nar/gku1153

**Published:** 2014-11-20

**Authors:** Jessica L. Wojtaszek, Su Wang, Hyungjin Kim, Qinglin Wu, Alan D. D'Andrea, Pei Zhou

**Affiliations:** 1 Department of Biochemistry, Duke University Medical Center, Durham, NC 27710, USA; 2 Department of Radiation Oncology, Dana-Farber Cancer Institute, Boston, MA 02215, USA

## Abstract

FAAP20 is an integral component of the Fanconi anemia core complex that mediates the repair of DNA interstrand crosslinks. The ubiquitin-binding capacity of the FAAP20 UBZ is required for recruitment of the Fanconi anemia complex to interstrand DNA crosslink sites and for interaction with the translesion synthesis machinery. Although the UBZ–ubiquitin interaction is thought to be exclusively encapsulated within the ββα module of UBZ, we show that the FAAP20–ubiquitin interaction extends beyond such a canonical zinc-finger motif. Instead, ubiquitin binding by FAAP20 is accompanied by transforming a disordered tail C-terminal to the UBZ of FAAP20 into a rigid, extended β-loop that latches onto the complex interface of the FAAP20 UBZ and ubiquitin, with the invariant C-terminal tryptophan emanating toward I44^Ub^ for enhanced binding specificity and affinity. Substitution of the C-terminal tryptophan with alanine in FAAP20 not only abolishes FAAP20–ubiquitin binding *in vitro*, but also causes profound cellular hypersensitivity to DNA interstrand crosslink lesions *in vivo*, highlighting the indispensable role of the C-terminal tail of FAAP20, beyond the compact zinc finger module, toward ubiquitin recognition and Fanconi anemia complex-mediated DNA interstrand crosslink repair.

## INTRODUCTION

Fanconi anemia (FA) is an autosomal recessive genetic disorder characterized by chromosomal instability, congenital abnormalities, early onset of cancer and high susceptibility to DNA cross-linking agents (1). FA patients have genetic mutations in one of 16 tumor suppressor genes that encode proteins functioning in the FA pathway to protect cells from genotoxic stress, including DNA interstrand crosslinks (ICLs) (2,3). At the center of the FA pathway is the recruitment of a multi-subunit FA core complex to sites of DNA damage. This complex, consisting of FANCA, FANCB, FANCC, FANCE, FANCF, FANCG, FANCL, FAAP20 and FAAP100, functions as an E3 ligase to monoubiquitinate the FANCD2-FANCI complex, signaling downstream DNA repair proteins involved in homologous recombination, nucleolytic excision and translesion synthesis (TLS) to orchestrate the repair of lesion DNA (4,5).

The function of the FA pathway, like other DNA repair pathways including TLS, relies heavily on the recognition of ubiquitinated substrates by ubiquitin-binding zinc finger (UBZ) domains within pathway proteins, such as those found in FAN1, FANCP (SLX4), Rad18, SNM1A and FAAP20 (6–8). FAAP20 is an integral component of the FA core complex and consists of an N-terminal FANCA-interacting region and a C-terminal UBZ domain. Its UBZ domain, consisting of a classical CCHC zinc-finger motif, has been shown to play an indispensable role for recruiting the FA core complex to ICL sites, for activating the FA pathway and for promoting the interaction of the FA core complex with PCNA-Rev1 DNA damage bypass complexes (9–12).

In this work, we report the solution structures of the UBZ domain of human FAAP20 and its complex with ubiquitin. We show that in contrast to the widely accepted notion of the compact ββα zinc finger module being the functional unit for ubiquitin recognition, FAAP20–ubiquitin interaction expands beyond the compact UBZ domain and requires the folding and interaction of the otherwise disordered C-terminal tail of FAAP20 for high-affinity binding. Further supporting this notion, we show alanine substitution of the absolutely conserved C-terminal tryptophan residue of FAAP20 abolishes ubiquitin binding and impairs FA core complex-mediated ICL repair *in vivo*.

## MATERIALS AND METHODS

### Protein constructs and cloning

The DNA sequence of residues 140–180 of human FAAP20 isoform 2 was synthesized; the polymerase chain reaction amplified DNA was double digested and ligated into a modified pET15b vector (EMD Biosciences, Inc.) between the NdeI and XhoI restriction sites. The final construct contained an N-terminal His_10_ tag, followed by a GB1 solubility enhancement tag (13,14). A tobacco etch virus (TEV) cleavage site was engineered between the GB1 tag and the FAAP20 sequence. The DNA sequence of human ubiquitin was cloned into the pET15b vector (EMD Biosciences, Inc.), with an N-terminal His_6_ tag and a thrombin cleavage site in between.

### Protein purification

The His_10_-GB1-fused FAAP20 construct and the His_6_-tagged human ubiquitin were overexpressed in *Escherichia coli* BL21 STAR (DE3) cells (Invitrogen). Bacterial cells were cultured in M9 minimal media using ^15^N-NH_4_Cl and ^13^C-glucose as the sole nitrogen and carbon sources (Cambridge Isotope Laboratories), and induced by IPTG (0.1 mM IPTG at 20°C for 18 h for His_10_-GB1-fused FAAP20 and 1 mM IPTG at 20°C for 18 h for human ubiquitin). A total of 50 μM ZnSO_4_ was added to the cell culture at the time of induction for overexpression of His_10_-GB1-fused FAAP20. The overexpressed proteins were purified by a Ni^2+^-NTA column; then the N-terminal His_6_-tag of human ubiquitin and the N-terminal His_10_-GB1 tag of the FAAP20 construct were removed by thrombin and TEV cleavage, respectively. A benzamidine column was used to remove thrombin, and a second Ni^2+^-NTA column was used to remove protein tags (His_6_-tag from ubiquitin and His_10_-GB1-tag from FAAP20) and the TEV protease. Both proteins were further purified by size-exclusion chromatography (Superdex 75, GE Healthcare). Nuclear magnetic resonance (NMR) samples of the apo FAAP20 UBZ and the FAAP20 UBZ–ubiquitin complex were exchanged into an NMR buffer containing 25 mM sodium phosphate, 100 mM KCl and 10% D_2_O or 100% D_2_O (pH 7.0) and concentrated to final protein concentrations of 0.8–3 mM.

### NMR spectroscopy

All NMR experiments were conducted at 25°C using Agilent INOVA 600 or 800 MHz spectrometers. NMR data were processed by NMRPipe (15) and scrub (16) and analyzed with SPARKY (17). Backbone resonances of apo FAAP20 and the FAAP20–ubiquitin complex were assigned by four pairs of sparsely sampled three-dimensional (3-D) triple-resonance experiments, and the sidechain resonances were assigned using the four-dimensional (4-D) sparsely sampled HC(co)NH-TOCSY and HCCH-TOCSY experiments.

For apo FAAP20, nuclear Overhauser effects (NOEs) identified from 3-D ^15^N-separated NOESY-HSQC, 4-D sparsely sampled ^13^C-HMQC-NOESY-^15^N-HSQC and ^13^C-HMQC-NOESY-HSQC experiments were used for automated cyana structure calculation (18) in the presence of dihedral angle constraints derived from talos+ analysis of chemical shift information (19). The final structural ensemble (10 structures) of apo FAAP20 displays no NOE violations > 0.5 Å and no dihedral angle violations > 5°. The statistics of the structural ensemble is shown in Supplementary Table SI.

For the FAAP20–ubiquitin complex, intermolecular NOEs were identified from the 4-D omit spectra as described previously (20). Intermolecular NOE crosspeaks were analyzed manually and converted into distance constraints using the calibration module in cyana (18). NOE crosspeaks from 3-D ^15^N-separated NOESY-HSQC and 4-D sparsely sampled ^13^C-HMQC-NOESY-^15^N-HSQC and ^13^C-HMQC-NOESY-HSQC experiments collected with a uniformly labeled complex sample were used for automated cyana structure calculation (18) in the presence of manually assigned intermolecular NOE constraints and dihedral angle constraints derived from talos+ analysis of chemical shift information (19). The final structural ensemble (10 structures) of the FAAP20 UBZ–ubiquitin complex displays no NOE violations > 0.5 Å and no dihedral angle violations > 5°. The statistics of the structural ensemble is shown in Supplementary Table SII.

Heteronuclear ^1^H–^15^N NOE experiments (21) were collected for 1 mM ^15^N-labeled FAAP20 in the absence (apo state) and in the presence (complex state) of 2 mM unlabeled ubiquitin. The ^1^H–^15^N heteronuclear NOE values were calculated as previously described (21).

### Isothermal titration calorimetry

Wild-type (WT) or mutant human ubiquitin (2–3 mM) was titrated into a solution of WT or mutant FAAP20 (0.2–0.3 mM) in a buffer containing 25 mM sodium phosphate, 100 mM KCl, pH 7.0. Twenty-eight injections of 10 μl each were performed at 25°C using a VP-ITC Microcalorimeter (GE Healthcare), and data were analyzed using the Origin software assuming one-site binding (Origin Lab).

### Cell culture and plasmid construction

U2OS and 293T cells were cultured in Dulbecco's modified Eagle's medium supplemented with 10% fetal bovine serum following standard culture conditions and procedures. Generation of FAAP20 constructs was described previously (9). Point mutations were introduced by QuikChange II XL Site-Directed Mutagenesis Kit (Agilent Technologies) and confirmed by DNA sequencing. Stable U2OS cells were generated by retroviral transduction of siRNA-resistant pMSCV-Flag-HA-FAAP20 variants followed by 2 μg/ml puromycin selection.

### Plasmid transfection and siRNA

Plasmid transfection for retroviral transduction was performed using Lipofectamine 2000 (Invitrogen) according to the manufacturer's protocols. siRNA duplexes were synthesized by Qiagen and transfected using Lipofectamine RNAiMAX (Invitrogen). The targeting sequence for FAAP20 is 5′-CACGGTGAGCCCGGAGCTGAT, and the nucleotides changed in the siRNA-resistant construct are shown in lower cases, 5′-gACtGTtAGtCCtGAaCTaAT.

### Protein analysis and antibodies

Cells were lysed with NETN300 buffer (300 mM NaCl, 0.2 mM EDTA, 50 mM Tris [pH 7.5], 1% NP40) supplemented with protease inhibitor cocktail (Roche). Cellular lysates were resolved by NuPAGE (Invitrogen) gels and transferred onto polyvinylidene fluoride (PVDF) membrane (EMD Millipore) followed by immunoblotting using antibodies as indicated: anti-FANCA (Bethyl), anti-FAAP20 (Sigma Atlas) and anti-Tubulin (Sigma). Signals were detected by either enhanced chemiluminescence method (Western Lightening, Perkin Elmer) or LAS-4000 Imaging system (GE Healthcare Life Sciences).

### Cytotoxicity assay

siRNA-treated U2OS cells were seeded on 96-well plates and treated with increasing doses of mitomycin C (MMC; Sigma) the following day. Cell viability was determined using the Cell Titer-Glo Luminescence Cell Viability Assay kit (Promega) and Spectramax M5 (Molecular Devices) 6 days following continued drug treatment.

## RESULTS

### The disordered C-terminal tail of FAAP20 UBZ is involved in ubiquitin binding

**Figure 1.**
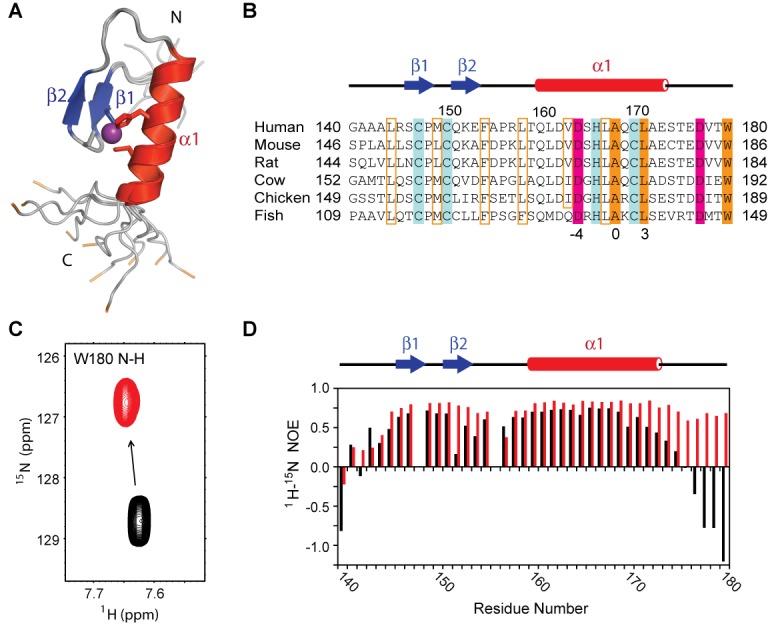
Solution structure of the human FAAP20 UBZ. (**A**) FAAP20 UBZ adopts a canonical ββα fold with the β-strands shown in blue, and α-helix shown in red. Sidechains of the zinc-coordinating CCHC motif are shown in the stick model and colored according to the secondary structures. The zinc atom is shown in the sphere model and colored in purple. The backbone of the terminal tryptophan, W180, is colored in orange. (**B**) Sequence alignment of the FAAP20 UBZ from different species. The conserved zinc-coordinating residues are highlighted in blue. Conserved residues involved in the formation of the hydrophobic core of the UBZ domain are boxed in orange and those involved in ubiquitin interaction are highlighted. Acidic residues are colored in pink and hydrophobic residues are colored in orange. (**C**) Titration of unlabeled ubiquitin into ^15^N-labeled FAAP20 significantly perturbs the backbone amide resonance of W180. Apo FAAP20 is shown in black and FAAP20 with excess ubiquitin in red. (**D**) ^1^H–^15^N heteronuclear NOEs plotted per residue of FAAP20. Values for the apo FAAP20 are shown with black bars and values for the FAAP20 with excess ubiquitin are shown with red bars.

In order to probe the molecular basis of ubiquitin recognition by the FAAP20 UBZ, we first determined the solution structure of the apo protein, consisting of human FAAP20 residues 140–180, by NMR (Figure 1A; statistics shown in Supplementary Table SI). Residues 144–173 of FAAP20 adopt a well-converged canonical ββα fold, with mean pairwise r.m.s. deviations of 0.22 and 1.11 Å for the backbone and heavy atoms, respectively. The ββα fold is held together by zinc coordination of conserved ‘finger’ residues, including C147 from β1, C150 from the ‘fingertip’ of the β1–β2 loop and H166 and C170 from the α-helix. Packing of the two β-strands against the α-helix is augmented by interactions among conserved hydrophobic residues, including L144 N-terminal to β1, M149 at the fingertip of the β1–β2 loop, F154 C-terminal to β2, L158 N-terminal to the α-helix, and V163 and L167 in the middle of the α-helix (Figure 1B). Outside of the compact ββα zinc-finger module, N-terminal residues 140–143 and C-terminal residues 174–180 are completely disordered.

Surprisingly, when we probed the FAAP20–ubiquitin interaction by NMR titration, we observed extensive resonance perturbation for residues of the disordered C-terminal tail in addition to residues of the ββα UBZ module. The backbone resonance of the terminal Trp residue (W180) of FAAP20, in particular, undergoes a prominent change of chemical shift upon ubiquitin binding (Figure 1C), indicating that it is experiencing a major alteration of the chemical environment and may likely be directly involved in ubiquitin binding. In order to further examine the potential involvement of the disordered C-terminal tail in ubiquitin binding, we conducted the ^1^H–^15^N heteronuclear NOE experiment, looking for changes in flexibility of the FAAP20 protein backbone upon ubiquitin binding (Figure 1D). In this experiment, a negative value of the heteronuclear NOE reflects rapid conformational fluctuation at the ps-to-ns timescale that is typically found in disordered loops, whereas a positive value reflects a lack of fast motion that is consistent with a rigid conformation commonly found in well-folded proteins (21). In the apo state, residues of the C-terminal tail of FAAP20 UBZ all displayed negative heteronuclear NOEs, consistent with the disordered conformation observed in the NMR ensemble of apo FAAP20. In contrast, these values became distinctly positive upon addition of 2-molar excess of ubiquitin, indicating that the C-terminal tail has gained rigidity upon the formation of the FAAP20–ubiquitin complex. Taken together, these data strongly support the involvement of the C-terminal tail of FAAP20, in addition to the canonical UBZ domain, in interaction with ubiquitin *in vitro*.

### Binding-induced folding of the FAAP20 C-terminal tail expands the canonical UBZ–ubiquitin interface

**Figure 2.**
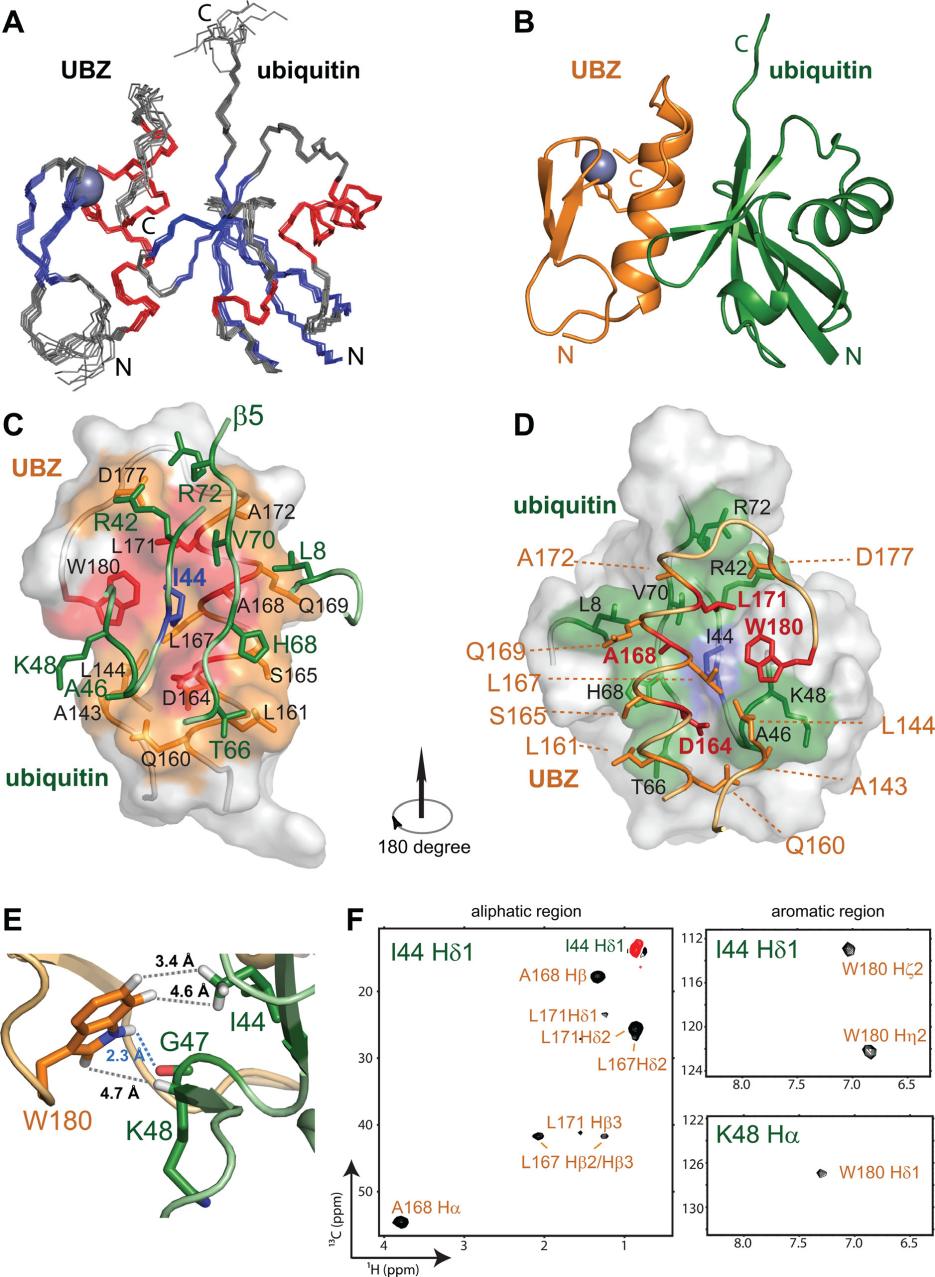
Solution structure of the human FAAP20–ubiquitin complex. (**A**) Backbone traces of the NMR ensemble of 10 structures. β-strands, α-helices and loops are colored in blue, red and gray, respectively. Zinc is shown as a gray sphere. (**B**) Ribbon diagram of the complex, with FAAP20 in orange and ubiquitin in green. (**C**) The surface representation of the FAAP20 UBZ with the interface colored in orange. The D–A–L motif and the C-terminal tryptophan residue are colored in red. The sidechains of the interfacial residues are shown in the stick model and labeled in black. Ubiquitin is colored in green with the sidechains of the interfacial residues shown in the stick model. The central residue I44^Ub^ is colored in blue. (**D**) The surface representation of ubiquitin with the interface colored in green. The central residue I44^Ub^ is colored in blue. The sidechains of the interfacial residues are shown in the stick model and labeled in black. The FAAP20 UBZ is colored in orange with the sidechains of the interfacial residues shown in the stick model. The D–A–L motif and the C-terminal tryptophan residue are colored in red. The two β-strands and the zinc ion are not shown for clarity. (**E**) Interactions between FAAP20 W180 (orange) and ubiquitin I44, G47 and K48 (green). The interacting sidechains are shown in the stick model. The hydrogen bond between W180 Hϵ1 and G47 backbone is shown as a blue dashed line. The hydrophobic interactions supported by intermolecular NOEs are shown as gray dashed lines. Distances of the interactions are labeled. (**F**) Representative 2-D slices of the 4-D ^13^C-HMQC-NOESY-HSQC omit spectra centered on I44 Hδ1 and K48 Hα, respectively, illustrating the intermolecular NOEs from FAAP20 to ubiquitin I44 Hδ1 and K48 Hα protons. Positive intermolecular NOE crosspeaks are colored in black and a negative diagonal peak is colored in red. Resonances of FAAP20 and ubiquitin are labeled in orange and green, respectively. The generation of omit spectra is shown in Supplementary Figure S1 in detail.

The unexpected involvement of the disordered C-terminal tail outside the FAAP20 UBZ for ubiquitin interaction is distinct from all other UBZ domains studied thus far (22–25) and warrants a thorough structural investigation. Using sparsely sampled 3-D and 4-D NMR spectroscopy (16,20), we have determined the solution structure of the FAAP20 UBZ–ubiquitin complex (Figure 2A and B), with the ensemble mean pairwise r.m.s. deviations of 0.55 and 1.16 Å for the backbone and heavy atoms, respectively (Supplementary Table SII).

Binding of FAAP20 causes little conformational change for ubiquitin, which adopts the familiar α/β-roll topology with the main α-helix wrapped in a distorted central β-sheet consisting of five strands (Figure 2B). At the opposite, solvent-exposed surface of the ubiquitin β-sheet lies a cluster of hydrophobic residues centered around I44^Ub^ for high-affinity interaction with FAAP20 (Figure 2C). In contrast, binding of FAAP20 to ubiquitin is accompanied by a major conformational switch, involving binding-induced attachment of the disordered C-terminal tail of apo FAAP20 to its compact ββα zinc finger module to form an expanded ubiquitin-binding interface (compare Figure 1A with Figure 2B and D).

The overall assembly of the FAAP20 UBZ–ubiquitin complex is similar to that of the MIU/IUIM–ubiquitin complex (26,27) and to the predicted structural model of the Pol η UBZ–ubiquitin complex (25), with the prominent UBZ helix packing against the solvent-exposed surface of the central β-sheet of ubiquitin. The FAAP20 UBZ helix is oriented in parallel with the central β-strand (β5) of ubiquitin, and the C-terminal end of the UBZ helix is located in close proximity to the C-terminus of ubiquitin (Figure 2C). At the center of the UBZ helix lies an invariant Ala residue (A168) that wedges into the hydrophobic pocket of ubiquitin encircled by L8^Ub^, I44^Ub^ and V70^Ub^. Along the same face of the FAAP20 helix, at one helical turn N-terminal to the central A168 (at the -4 position in the primary sequence, Figure 1B) lies a conserved Asp residue (D164) that forms hydrogen bonds with the backbone of A46^Ub^ and G47^Ub^, anchoring the N-terminal half of the UBZ helix to ubiquitin. Such a helix–ubiquitin interaction is augmented by hydrophobic interactions at the C-terminal half of the UBZ helix, with the invariant hydrophobic residue L171 located at one helical turn C-terminal to A168 (at the +3 position, Figure 1B) along the same face of the UBZ helix to interact with I44^Ub^, V70^Ub^ and the side chain of R42^Ub^, further enhancing the binding toward ubiquitin. A group of less conserved residues along the UBZ helix also contribute to the human FAAP20 interaction with ubiquitin, including S165 that forms a hydrogen bond with H68^Ub^, the sidechain of Q169 that interacts with L8^Ub^ and A172 that interacts with V70^Ub^ (Figure 2D).

Unique to the FAAP20 UBZ–ubiquitin interface is the presence of binding-induced folding of a disordered C-terminal tail of FAAP20 containing two invariant residues: Asp (D177) and Trp (W180) (Figure 1B). Although residues of this tail are highly dynamic in the apo state as reflected by their negative heteronuclear NOE values (Figure 1D), they form an extended β-loop that is fixated by joint interactions with ubiquitin and with the core module of the FAAP20 UBZ. In particular, the sidechain of D177 points toward the sidechains of R42^Ub^ and R72^Ub^, potentially latching onto ubiquitin through salt bridges with these residues. W180, the Trp residue at the very C-terminus of this tail in FAAP20, appears to play a crucial role in mediating FAAP20–ubiquitin binding. Emanating toward I44^Ub^ from the now extended C-terminal loop of FAAP20 that stretches along the UBZ–ubiquitin interface, the indole group of W180 is affixed to the carbonyl group of G47^Ub^ located within the β3–β4 loop of ubiquitin through a hydrogen bond of the W180 imino group (Figure 2E). Such an interaction is supported by the observed intermolecular NOE between the W180 aromatic proton Hδ1 and the Hα proton of K48^Ub^ (Figure 2F, lower right panel). The W180 indole ring points toward I44^Ub^ and is juxtaposed between the sidechains of K48^Ub^ and Q49^Ub^ of the β3–β4 loop of ubiquitin, and P148 and M149 of the fingertip and L167 of the central helix of the FAAP20 UBZ. Importantly, W180, together with a cluster of conserved residues of the compact zinc finger module of FAAP20, including L167, A168 and L171, forms an encircled hydrophobic pocket around I44^Ub^ for high-affinity ubiquitin binding (Figure 2C and D). Accordingly, numerous intermolecular NOEs are observed in the 4-D difference NOE (omit) spectrum between I44^Ub^ and surrounding FAAP20 residues (Figure 2F and Supplementary Figure S1). Highlighting the integral structure of the FAAP20 C-terminal tail in ubiquitin recognition, residues of this tail all display positive heteronuclear NOEs that are congruent with FAAP20 residues of the ββα zinc-finger module (Figure 1D).

### The conserved D–A–L motif of the FAAP20 UBZ helix contributes to ubiquitin binding

After determining the solution structure of the FAAP20 UBZ–ubiquitin complex, we investigated the FAAP20–ubiquitin interaction by isothermal titration calorimetry (ITC) measurements in order to probe the contribution of individual residues to the binding affinity (Table 1, Supplementary Figure S2). Titration of WT ubiquitin into WT FAAP20 UBZ revealed a *K*_d_ value of 9.26 μM, a binding affinity stronger than the affinities of most ubiquitin-binding domain (UBD)–ubiquitin interactions.

**Table 1.** Binding affinities of the human FAAP20 UBZ–ubiquitin complexes measured by ITC

FAAP20 UBZ	Ubiquitin	*K*_d_ (μM)
WT	WT	9.26
WT	I44A	NDB
D164A	WT	NDB
A168Y	WT	NDB
L171A	WT	529
W180A	WT	NDB

NDB: No detectable binding or too weak to fit reliably (*K*_d_ > 600 μM).

Among the interface residues of the FAAP20–ubiquitin complex, I44^Ub^ is at the center of the FAAP20 UBZ–ubiquitin interaction and displays numerous intermolecular NOEs to FAAP20 residues, including W180 of the C-terminal tail (Figure 2F). Unsurprisingly, mutation of I44^Ub^ to Ala completely abolished the ubiquitin binding by FAAP20, verifying that ubiquitin is being recognized through the canonical hydrophobic patch centered at I44^Ub^. On the FAAP20 side, A168 is located at the center of the UBZ helix and plays a pivotal role in anchoring the UBZ helix to the conserved hydrophobic pocket of ubiquitin formed by L8^Ub^, I44^Ub^ and V70^Ub^. Accordingly, substitution of A168 by Tyr eliminated the FAAP20–ubiquitin interaction. Point mutation of D164A of the FAAP20 UBZ similarly disrupted ubiquitin binding in our ITC studies, consistent with a previous report (12). Finally, mutation of L171A significantly weakened the FAAP20 interaction with ubiquitin. Taken together, these observations further support the structurally observed binding mode of the FAAP20–ubiquitin complex, corroborating the recognition mode of the D–A–L motif along the surface of the UBZ α-helix for ubiquitin interaction.

### The terminal tryptophan is required for FAAP20–ubiquitin binding *in vitro* and efficient ICL repair *in vivo*

**Figure 3.**
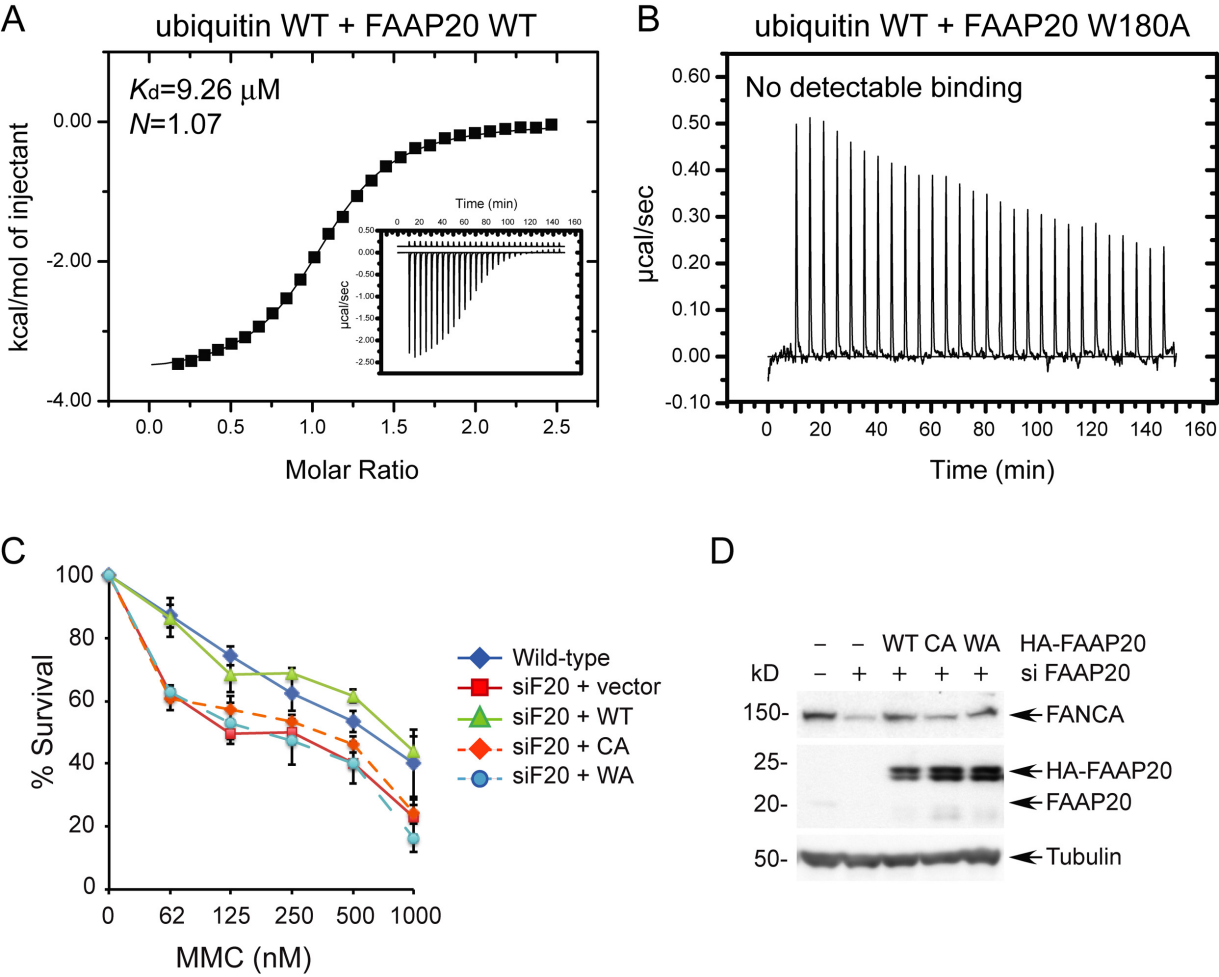
The C-terminal tryptophan residue (W180) of FAAP20 outside the compact UBZ module plays an indispensable role in ubiquitin recognition by FAAP20 *in vitro* and in efficient ICL DNA repair *in vivo*. (**A**) Measurement of binding affinity between WT FAAP20 and WT ubiquitin by ITC. (**B**) Mutation of W180A in FAAP20 abolished ubiquitin binding in ITC measurements. (**C**) U2OS cells stably expressing siRNA-resistant FAAP20 wild-type, C147A and C150A (CA), or W180A (WA) were transfected with siRNA against FAAP20 (siF20) for 48 h, and cell viability was determined 6 days after the treatment of indicated doses of mitomycin C. (**D**) Immunoblot analysis of U2OS cells in (C) harvested 72 h after siRNA treatment.

Since the FAAP20–ubiquitin interaction uniquely features an expanded ubiquitin-binding interface beyond the compact ββα zinc-finger module, with the absolutely conserved Trp residue (W180) of the disordered C-terminal tail of apo FAAP20 participating in numerous interactions with ubiquitin residues in the protein complex, including I44^Ub^ at the center of the ubiquitin interface, we conducted *in vitro* and *in vivo* experiments to evaluate the consequence of a W180A mutation in affecting the FAAP20–ubiquitin interaction and in FA core complex-mediated ICL repair. In order to make sure that the W180A mutation does not alter the UBZ structure, a ^1^H–^15^N HSQC spectrum of the FAAP20 W180A mutant was collected (Supplementary Figure S3). With the exception of the few C-terminal residues neighboring W180 in the primary sequence, the overall spectra of the WT FAAP20 UBZ and the W180A mutant are nearly superimposable, consistent with the notion of a disordered C-terminal tail in apo FAAP20 and verifying that the W180A mutation does not disturb the ββα fold of the UBZ. Surprisingly, in contrast to the tight binding of WT FAAP20 toward ubiquitin in ITC measurements (Figure 3A), the W180A mutant completely abolished the FAAP20 interaction with ubiquitin (Figure 3B), revealing that W180 not only contributes to, but is also required for ubiquitin binding *in vitro*.

In order to assess the contribution of the C-terminal tryptophan residue in regulating DNA repair *in vivo*, we also tested the ability of full-length FAAP20 harboring a W180A mutation to complement the DNA damage sensitivity of the FAAP20-depleted mammalian cells. The functional integrity of the FAAP20 UBZ is required for conferring cellular resistance to DNA ICL-inducing agent, MMC (12). Therefore, we replaced endogenous FAAP20 depleted by siRNA with siRNA-resistant FAAP20 variants and challenged cells with MMC. Stable expression of siRNA-resistant WT FAAP20 could restore the hypersensitivity to MMC caused by FAAP20 depletion, while neither C147A & C150A (the mutations disrupting the zinc finger fold) nor the W180A mutant could rescue the DNA damage sensitization phenotype (Figure 3C and D). Such an observation further emphasizes the functional importance of W180 and the C-terminal tail outside the core UBZ domain in mediating the FAAP20 interaction with ubiquitin, which is essential for the ICL DNA repair *in vivo*. It is intriguing to note that FAAP20 has eight isoforms, but only isoforms 2 and 6 contain an intact UBZ domain with a C-terminal Trp residue and hence are capable of interaction with ubiquitin and execution of ICL DNA repair. The functions of the other isoforms, however, await to be determined.

## DISCUSSION

**Figure 4.**
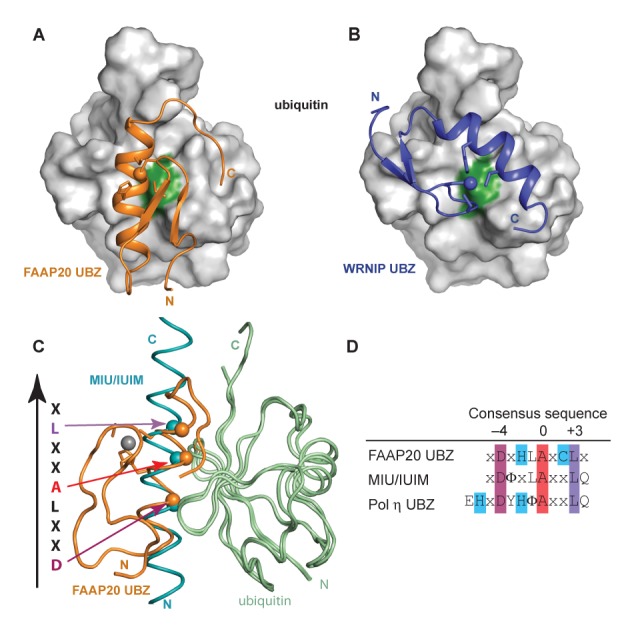
Ubiquitin recognition by the human FAAP20 UBZ helix is distinct from that of the WRNIP UBZ, but shows similarity to that of MIU/IUIM. Panels (**A**) and (**B**) show the ribbon diagrams of the FAAP20–ubiquitin complex (orange, this work) and the WRNIP UBZ–ubiquitin complex (blue, PDB 3VHT), respectively. Ubiquitin is shown in identical orientation. The surface of ubiquitin is colored in gray with the central interfacial residue I44 colored in green. (**C**) Overlay of the human FAAP20 UBZ (orange)–ubiquitin (pale green) complex (this work) with the MIU/IUIM (cyan)–ubiquitin (pale green) complex (PDB 2FIF). The conserved D(−4)-A(0)-L(+3) residues along the α-helix are shown in sphere and indicated by arrows colored according to the sequence alignment in (D). (**D**) Alignment of the consensus sequence of the FAAP20 UBZ helix with those of the MIU/IUIM and Pol η UBZ helices. The central invariant alanine is highlighted in red, the conserved aspartate at the −4 position in magenta, the conserved leucine at the +3 position in purple and the zinc ligands in blue.

Depending on the organization of the zinc-coordinating residues and ubiquitin-binding motifs in the primary sequence, UBZ domains are classified into different subgroups, including the UBZ3 type of zinc fingers consisting of CCHH zinc-coordinating residues represented by the Pol η UBZ, and the UBZ4 type of zinc fingers consisting of CCHC zinc-coordinating residues that are found in a variety of proteins involved in DNA repair activities. Structural comparison of the FAAP20 UBZ–ubiquitin complex to the WRNIP UBZ–ubiquitin complex (PDB 3VHT), which shares a similar ubiquitin-binding interface with the recently reported Rad18 UBZ–ubiquitin complex (22), reveals a completely different orientation of the central UBZ helix, even though the FAAP20 UBZ is sometimes referred to as a UBZ4 domain (9,11), the same type as the UBZs of WRNIP and Rad18 (Figure 4A and B). Further structural comparison shows ubiquitin recognition by the FAAP20 UBZ helix is actually similar to that of the MIU/IUIM helix in the ubiquitin-bound complex (26,27), a binding mode that features a D(−4)-A(0)-L(+3) motif along the ubiquitin-recognition α-helix and is also shared by the Pol η UBZ, a UBZ3 type of zinc finger (25) (Figure 4C and D). The structural observation of a distinct ubiquitin-binding mode of the FAAP20 UBZ from that of the UBZ4 type zinc finger lends further credence to the suggestion of the FAAP20 UBZ as the founding member of the UBZ2 family that is characterized by the CCHC zinc-coordinating residues and the DxHxAxCL motif (28).

All of the UBZ fingers that have been characterized thus far interact with ubiquitin exclusively through the compact ββα zinc finger module. FAAP20 also contains a well-defined UBZ, together with a C-terminal tail that is disordered in the absence of ubiquitin. Unexpectedly, the structural elucidation of the FAAP20–ubiquitin complex reveals a binding-induced folding of the disordered C-terminal tail of FAAP20 that expands the ubiquitin-binding interface beyond the canonical UBZ module for high-affinity interaction with ubiquitin. In particular, the very C-terminal Trp residue (W180), upon transforming the disordered tail of FAAP20 into an extended β-loop, buries its sidechain into the interface of the FAAP20 UBZ and ubiquitin to fortify the FAAP20–ubiquitin interaction. The FAAP20 mutant with alanine substitution of the terminal Trp residue (W180A) abolishes ubiquitin binding *in vitro* and ICL DNA repair activity *in vivo*, emphasizing the crucial contribution of the FAAP20 C-terminal tail to FAAP20 function and highlighting a unique binding mode not observed in any of the previously characterized UBZ–ubiquitin complexes.

## ACCESSION NUMBERS

The structures of apo FAAP20 and the FAAP20–ubiquitin complex have been deposited in RCSB, with the accession codes of 2muq and 2mur, respectively. The chemical shift assignments of apo FAAP20 and the FAAP20–ubiquitin complex have been deposited in BMRB, with the accession codes of 25229 and 25230, respectively.

## SUPPLEMENTARY DATA

Supplementary Data are available at NAR Online.

SUPPLEMENTARY DATA

## References

[1] D'Andrea A.D. (2010). Susceptibility pathways in Fanconi's anemia and breast cancer. N. Engl. J. Med..

[2] Kottemann M.C., Smogorzewska A. (2013). Fanconi anaemia and the repair of Watson and Crick DNA crosslinks. Nature.

[3] Walden H., Deans A.J. (2014). The Fanconi anemia DNA repair pathway: structural and functional insights into a complex disorder. Annu. Rev. Biophys..

[4] Kim H., D'Andrea A.D. (2012). Regulation of DNA cross-link repair by the Fanconi anemia/BRCA pathway. Genes Dev..

[5] Huang Y., Leung J.W., Lowery M., Matsushita N., Wang Y., Shen X., Huong D., Takata M., Chen J., Li L. (2014). Modularized functions of the Fanconi anemia core complex. Cell. Rep..

[6] Garner E., Smogorzewska A. (2011). Ubiquitylation and the Fanconi anemia pathway. FEBS Lett..

[7] Liu T., Ghosal G., Yuan J., Chen J., Huang J. (2010). FAN1 acts with FANCI-FANCD2 to promote DNA interstrand cross-link repair. Science.

[8] Bienko M., Green C.M., Crosetto N., Rudolf F., Zapart G., Coull B., Kannouche P., Wider G., Peter M., Lehmann A.R. (2005). Ubiquitin-binding domains in Y-family polymerases regulate translesion synthesis. Science.

[9] Kim H., Yang K.L., Dejsuphong D., D'Andrea A.D. (2012). Regulation of Rev1 by the Fanconi anemia core complex. Nat. Struct. Mol. Biol..

[10] Yan Z., Guo R., Paramasivam M., Shen W., Ling C., Fox D., Wang Y., Oostra A.B., Kuehl J., Lee D.Y. (2012). A ubiquitin-binding protein, FAAP20, links RNF8-mediated ubiquitination to the Fanconi anemia DNA repair network. Mol. Cell.

[11] Leung J.W., Wang Y., Fong K.W., Huen M.S., Li L., Chen J. (2012). Fanconi anemia (FA) binding protein FAAP20 stabilizes FA complementation group A (FANCA) and participates in interstrand cross-link repair. Proc. Natl. Acad. Sci. U.S.A..

[12] Ali A.M., Pradhan A., Singh T.R., Du C., Li J., Wahengbam K., Grassman E., Auerbach A.D., Pang Q., Meetei A.R. (2012). FAAP20: a novel ubiquitin-binding FA nuclear core-complex protein required for functional integrity of the FA-BRCA DNA repair pathway. Blood.

[13] Zhou P., Lugovskoy A.A., Wagner G. (2001). A solubility-enhancement tag (SET) for NMR studies of poorly behaving proteins. J. Biomol. NMR.

[14] Zhou P., Wagner G. (2010). Overcoming the solubility limit with solubility-enhancement tags: successful applications in biomolecular NMR studies. J. Biomol. NMR.

[15] Delaglio F., Grzesiek S., Vuister G.W., Zhu G., Pfeifer J., Bax A. (1995). NMRPipe: a multidimensional spectral processing system based on UNIX pipes. J. Biomol. NMR.

[16] Coggins B.E., Werner-Allen J.W., Yan A., Zhou P. (2012). Rapid protein global fold determination using ultrasparse sampling, high-dynamic range artifact suppression, and time-shared NOESY. J. Am. Chem. Soc..

[17] Goddard T.D., Kneller D.G. (2008). SPARKY 3.

[18] Güntert P. (2004). Automated NMR structure calculation with CYANA. Methods Mol. Biol..

[19] Shen Y., Delaglio F., Cornilescu G., Bax A. (2009). TALOS+: a hybrid method for predicting protein backbone torsion angles from NMR chemical shifts. J. Biomol. NMR.

[20] Wang S., Zhou P. (2014). Sparsely-sampled, high-resolution 4-D omit spectra for detection and assignment of intermolecular NOEs of protein complexes. J. Biomol. NMR.

[21] Kay L.E., Torchia D.A., Bax A. (1989). Backbone dynamics of proteins as studied by 15N inverse detected heteronuclear NMR spectroscopy: application to staphylococcal nuclease. Biochemistry.

[22] Rizzo A.A., Salerno P.E., Bezsonova I., Korzhnev D.M. (2014). NMR structure of the human Rad18 zinc finger in complex with ubiquitin defines a class of UBZ domains in proteins linked to the DNA damage response. Biochemistry.

[23] Cordier F., Grubisha O., Traincard F., Veron M., Delepierre M., Agou F. (2009). The zinc finger of NEMO is a functional ubiquitin-binding domain. J. Biol. Chem..

[24] Ceregido M.A., Spinola Amilibia M., Buts L., Rivera-Torres J., Garcia-Pino A., Bravo J., van Nuland N.A. (2014). The structure of TAX1BP1 UBZ1+2 provides insight into target specificity and adaptability. J. Mol. Biol..

[25] Bomar M.G., Pai M.T., Tzeng S.R., Li S.S., Zhou P. (2007). Structure of the ubiquitin-binding zinc finger domain of human DNA Y-polymerase eta. EMBO Rep..

[26] Lee S., Tsai Y.C., Mattera R., Smith W.J., Kostelansky M.S., Weissman A.M., Bonifacino J.S., Hurley J.H. (2006). Structural basis for ubiquitin recognition and autoubiquitination by Rabex-5. Nat. Struct. Mol. Biol..

[27] Penengo L., Mapelli M., Murachelli A.G., Confalonieri S., Magri L., Musacchio A., Di Fiore P.P., Polo S., Schneider T.R. (2006). Crystal structure of the ubiquitin binding domains of rabex-5 reveals two modes of interaction with ubiquitin. Cell.

[28] Hofmann K. (2009). Ubiquitin-binding domains and their role in the DNA damage response. DNA Repair.

