# Supplementary material for: Ubiquitin recognition by FAAP20 expands the complex interface beyond the canonical UBZ domain
Source: Nucleic Acids Res. 2014 Nov 20;42(22):13997–4005. doi: 10.1093/nar/gku1153 (PMC4267625; doi:10.1093/nar/gku1153)
Supplement: SUPPLEMENTARY DATA [file supp_42_22_13997__index.html]

Ubiquitin recognition by FAAP20 expands the complex interface beyond the canonical UBZ domain — Ubiquitin recognition by FAAP20 expands the complex interface beyond the canonical UBZ domain — SUPPLEMENTARY DATA 

# Ubiquitin recognition by FAAP20 expands the complex interface beyond the canonical UBZ domain

## SUPPLEMENTARY DATA

**Files in this Data Supplement:**

- SUPPLEMENTARY DATA
