# Supplementary material for: Ubiquitin recognition by FAAP20 expands the complex interface beyond the canonical UBZ domain
Source: Nucleic Acids Res. 2014 Nov 20;42(22):13997–4005. doi: 10.1093/nar/gku1153 (PMC4267625; doi:10.1093/nar/gku1153)
Supplement: SUPPLEMENTARY DATA [file supp_gku1153_nar-02777-d-2014-File006.pdf]

Supplemental Information for:

Ubiquitin Recognition by FAAP20 Expands the Complex Interface Beyond the Canonical UBZ Domain

Jessica L. Wojtaszek[1,4], Su Wang[1,4], Hyungjin Kim[2,3], Qinglin Wu[1], Alan D. D'Andrea[2], Pei Zhou[1,*]

[1]Department of Biochemistry, Duke University Medical Center, Durham, North Carolina 27710, USA

[2]Department of Radiation Oncology, Dana-Farber Cancer Institute, Boston, Massachusetts 02215, USA

[3]Current Address: Department of Pharmacological Sciences, Stony Brook University, Stony Brook, NY 11794-8651, USA

[4]The authors wish it to be known that, in their opinion, the first two authors should be regarded as joint First Authors.

* To whom correspondence should be addressed. Tel: +1 919-668-6409; Fax: 919-684-8885; E-mail: peizhou@biochem.duke.edu

Table of Contents:

Table SI -- **S2**

Table SII -- **S3**

Figure S1 -- **S4**

Figure S2 -- **S5**

Figure S3 -- **S6**

References -- **S7**

TABLE SI

Structural statistics for the human FAAP20 UBZ (10 structures)[a]

FAAP20 UBZ (140-180)	
NOE distance restraints	852
Intra-residue	144
Sequential	246
Medium-range ($1< \|i-j\| \leq 4$)	278
Long-range ($\|i-j\| \geq 5$)	184
Hydrogen bond constraints[b]	28
Dihedral angle constraints[c]	44
Target function value	1.04 ± 0.01
Ramachandran plot[d]	
Favored region (98%)	91.9
Allowed region (>99.8%)	100.0
Mean pairwise RMSD (FAAP20 UBZ 144-173)	
Backbone	0.29 ± 0.07 Å
Heavy Atoms	1.11 ± 0.18 Å

[a] None of these structures exhibit distance violations greater than 0.5 Å or dihedral angle violations greater than 5°.

[b] Two constraints ($d_{HN\text{-}O} \leq 2.5$ Å and $d_{N\text{-}O} \leq 3.5$ Å) are used for each identified hydrogen bond.

[c] Dihedral angle constraints were generated by TALOS+ based on backbone atom chemical shifts (1), and by analysis of NOE patterns.

[d] MOLPROBITY was used to assess the quality of the structures (2).

TABLE SII

Structural statistics for the human FAAP20 UBZ-ubiquitin complex (10 structures)[a]	
FAAP20 UBZ (140-180)	
NOE distance restraints	1028
Intra-residue	126
Sequential	291
Medium-range (1< |i-j| ≤4)	309
Long-range (|i-j| ≥ 5)	302
Hydrogen bond constraints[b]	28
Dihedral angle constraints[c]	55
Ubiquitin (1-76)	
NOE distance restraints	2867
Intra-residue	328
Sequential	616
Medium-range (1< |i-j| ≤4)	569
Long-range (|i-j| ≥ 5)	1354
Hydrogen bond constraints[b]	60
Dihedral angle constraints[c]	132
Intermolecular NOE distance constraints	187
Target function value	2.38 ± 0.09
Ramachandran plot[d]	
Favored region (98%)	90.8
Allowed region (>99.8%)	99.3
Mean pairwise RMSD (FAAP20 UBZ 143-180; Ubiquitin 1-74)	
Backbone	0.55 ± 0.08 Å
Heavy Atoms	1.16 ± 0.08 Å

[a] None of these structures exhibit distance violations greater than 0.5 Å or dihedral angle violations greater than 5°.

[b] Two constraints ($d_{HN\text{-}O}$ ≤ 2.5 Å and $d_{N\text{-}O}$ ≤ 3.5 Å) are used for each identified hydrogen bond.

[c] Dihedral angle constraints were generated by TALOS+ based on backbone atom chemical shifts (1), and by analysis of NOE patterns.

[d] MOLPROBITY was used to assess the quality of the structures (2).

SUPPLEMENTAL INFORMATION FIGURES

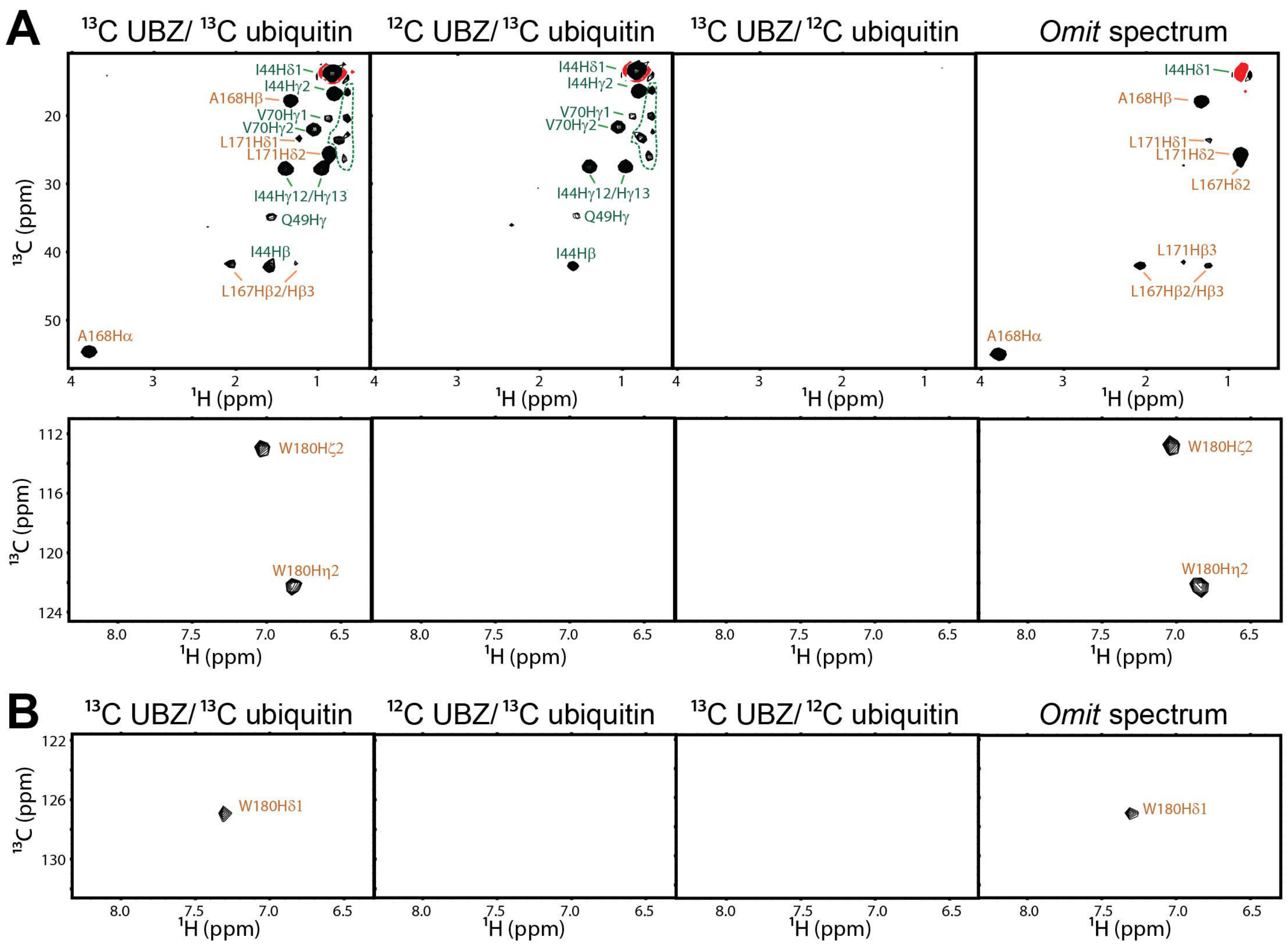

Figure S1. Intermolecular NOE difference (omit) spectrum of the human FAAP20-ubiquitin complex. Sparsely-sampled 4-D ^{13}C-HMQC–NOESY–HSQC spectra were collected for the FAAP20-ubiquitin complex with both components or with individual components ^{13}C-labeled as described previously (3). Reconstruction of the difference time domain signals of the uniformly labeled protein complex from component-labeled samples generated an omit spectrum containing only intermolecular NOEs. Slight over-subtraction of time domain data from individual components generates negative diagonal signals (*red*) in the omit spectrum and ensures that all of the positive crosspeaks originate from intermolecular NOEs. Panel (A) (upper, aliphatic regions; lower, aromatic regions) shows sections of F1-F2 slices of the corresponding 4-D spectra centered at 13.52 ppm in F3 and 0.796 ppm in F4, displaying NOEs to I44^{Ub} Hδ1. Peaks circled by dash lines are off-plane signals. Panel (B) shows sections of F1-F2 slices of the corresponding 4-D spectra centered at 53.88 ppm in F3 and 4.764 ppm in F4 in the aromatic region, displaying NOEs to the ubiquitin K48 Hα.

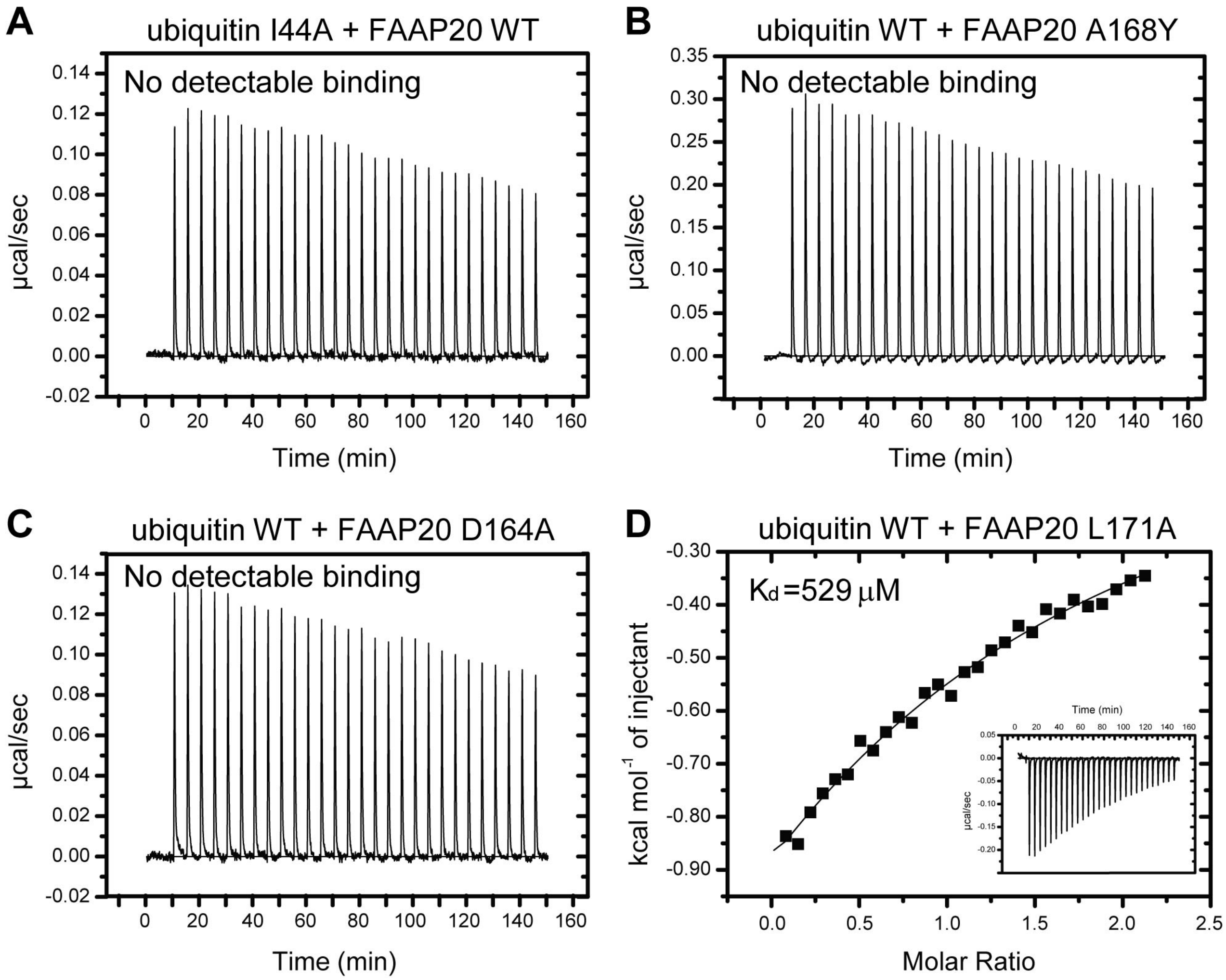

Figure S2. Mutations of the interface residues affect the FAAP20-ubiquitin binding. Raw data of heat change per injection and the fitted affinity curve, whenever applicable, are shown for (A) the ubiquitin I44A mutant, (B) the FAAP20 A168Y mutant, (C) the FAAP20 D164A mutant, and (D) the FAAP20 L171A mutant, respectively.

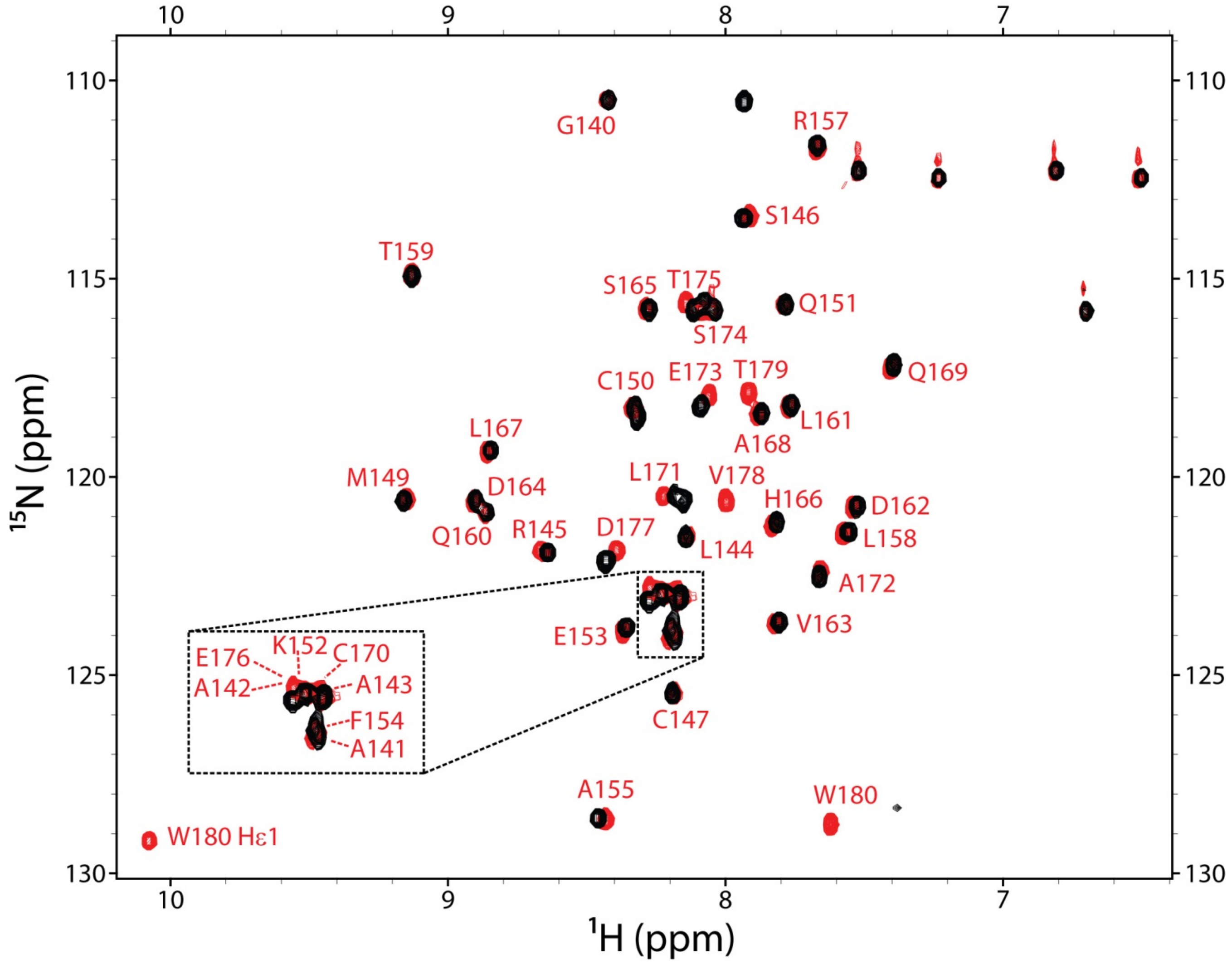

Figure S3. ^{1}H-^{15}N HSQC spectra of the human FAAP20 UBZ WT protein (red) and W180A mutant (black).

Supplemental Information REFERENCES

1. Shen, Y., Delaglio, F., Cornilescu, G. and Bax, A. (2009) TALOS+: a hybrid method for predicting protein backbone torsion angles from NMR chemical shifts. *Journal of biomolecular NMR*, **44**, 213-223.

2. Davis, I.W., Leaver-Fay, A., Chen, V.B., Block, J.N., Kapral, G.J., Wang, X., Murray, L.W., Arendall, W.B., 3rd, Snoeyink, J., Richardson, J.S. *et al.* (2007) MolProbity: all-atom contacts and structure validation for proteins and nucleic acids. *Nucleic Acids Res.*, **35**, W375-383.

3. Wang, S. and Zhou, P. (2014) Sparsely-sampled, high-resolution 4-D omit spectra for detection and assignment of intermolecular NOEs of protein complexes. *Journal of biomolecular NMR*, **59**, 51-56.
